# Validation of a Lower Back “Wearable”-Based Sit-to-Stand and Stand-to-Sit Algorithm for Patients With Parkinson's Disease and Older Adults in a Home-Like Environment

**DOI:** 10.3389/fneur.2018.00652

**Published:** 2018-08-10

**Authors:** Minh H. Pham, Elke Warmerdam, Morad Elshehabi, Christian Schlenstedt, Lu-Marie Bergeest, Maren Heller, Linda Haertner, Joaquim J. Ferreira, Daniela Berg, Gerhard Schmidt, Clint Hansen, Walter Maetzler

**Affiliations:** ^1^Department of Neurology, University Hospital Schleswig-Holstein, Kiel University, Kiel, Germany; ^2^Digital Signal Processing and System Theory, Faculty of Engineering, Kiel University, Kiel, Germany; ^3^Department of Neurodegeneration, Center for Neurology, Hertie Institute for Clinical Brain Research, University of Tübingen, Tübingen, Germany; ^4^DZNE, German Center for Neurodegenerative Diseases, Tübingen, Germany; ^5^Clinical Pharmacology Unit, Instituto de Medicina Molecular, Lisbon, Portugal; ^6^Laboratory of Clinical Pharmacology and Therapeutics, Faculty of Medicine, University of Lisbon, Lisbon, Portugal

**Keywords:** accelerometer, gyroscope, home-like activities, older adults, PD patients, postural transition

## Abstract

**Introduction:** Impaired sit-to-stand and stand-to-sit movements (postural transitions, PTs) in patients with Parkinson's disease (PD) and older adults (OA) are associated with risk of falling and reduced quality of life. Inertial measurement units (IMUs, also called “wearables”) are powerful tools to monitor PT kinematics. The purpose of this study was to develop and validate an algorithm, based on a single IMU positioned at the lower back, for PT detection and description in the above-mentioned groups in a home-like environment.

**Methods:** Four PD patients (two with dyskinesia) and one OA served as algorithm training group, and 21 PD patients (16 without and 5 with dyskinesia) and 11 OA served as test group. All wore an IMU on the lower back and were videotaped while performing everyday activities for 90–180 min in a non-standardized home-like environment. Accelerometer and gyroscope signals were analyzed using discrete wavelet transformation (DWT), a six degrees-of-freedom (DOF) fusion algorithm and vertical displacement estimation.

**Results:** From the test group, 1,001 PTs, defined by video reference, were analyzed. The accuracy of the algorithm for the detection of PTs against video observation was 82% for PD patients without dyskinesia, 47% for PD patients with dyskinesia and 85% for OA. The overall accuracy of the PT direction detection was comparable across groups and yielded 98%. Mean PT duration values were 1.96 s for PD patients and 1.74 s for OA based on the algorithm (*p* < 0.001) and 1.77 s for PD patients and 1.51 s for OA based on clinical observation (*p* < 0.001).

**Conclusion:** Validation of the PT detection algorithm in a home-like environment shows acceptable accuracy against the video reference in PD patients without dyskinesia and controls. Current limitations are the PT detection in PD patients with dyskinesia and the use of video observation as the video reference. Potential reasons are discussed.

## Introduction

Falls are dangerous incidents often occurring at home for older adults (OA) resulting in injury, and consequently decreased quality of life (1–3). A positive history of falls increases fear of falling, further contributing to future falls (1, 4). Movement deficits often occur in neurological diseases. Within this spectrum Parkinson's disease (PD) is a predominantly motor disorder and patients with PD are specifically prone to increased fall risk (5). In both OA and PD patients, falls frequently happen during sit-to-stand and stand-to-sit movements (postural transitions, PTs), i.e., during changes of posture that require multi-limb coordination (6–8). Usually the diagnosis of these deficits at the doctor's office or in hospitals are based on qualitative parameters or on semi-quantitative scoring tools. The Unified Parkinson Disease Rating Scale (MDS-UPDRS) is one of many tools to rate motor symptoms including gait and postural stability in PD (9). Such tools have been subject to multiple validation studies and reflect disease state relatively well, but a downside is the large inter-rater variability and subjectivity (10–13). Over the last decade, inertial measurement units (IMUs, also called “wearables”), force plates (14), and complex optical 3D motion capture systems have been developed making it interesting for medical purposes (15–25) especially in the complementary assessment of gait and balance (25, 26). A body of literature describes assessment technologies and algorithms for the detection of PTs (27–31). While some systems are expensive and restricted to the laboratory (i.e., force plates, 3D motion capture), IMUs are a good trade-off. IMUs are microelectromechanical systems with multiple degrees of freedom (DOF; e.g., 3D accelerometers, 3D gyroscopes and 3D magnetometers). They are advantageously priced, light-weight, and can measure at frequencies sufficient to capture even fast human movements (32, 33). An additional argument for IMUs is their applicability in virtually any environment especially outside the clinic.

Assessments performed within the clinical environment or a laboratory setting reflect only parts of human behavior. For example, these assessments often measure unintentional (i.e., non-targeted) movements. Assessment of purposeful and target-oriented movements under unsupervised conditions can add highly relevant and complementary insight into human movements and mobility, including treatment effects (34–37). Therefore, a substantial interest is coming from legal institutions, such as the European Medicines Agency (EMA), the Food and Drug Administration (FDA), and from pharmaceutic companies to include parameters collected in the real life environment of study participants.

IMU-based PT studies have shown that PTs differ between OA and PD patients (38) and that PT characteristics change when the disease progresses (39). These studies have most often been performed under standardized lab situations, i.e., PT movements were pre-defined and instructed. However, the behavior in real-life environment may be even more relevant for clinical judgment (34). Therefore, algorithms that have been developed based on lab-based assessments may not be suitable for home assessments. They obviously do not address the high variance of PTs and increase the risk of false-positive (i.e., the detection of movements that are no PTs) and false-negative PTs (i.e., the non-detection of actual PTs) under daily living conditions.

In this study, we developed and validated a PT detection algorithm in PD patients and OA that performed purposeful movements in a home-like environment, from data of a lower back-worn IMU.

## Methods

### Study participants, setting and data collection process

The study was approved by the ethical committee of the Medical Faculty of the University of Tübingen (protocol number 399/2012BO2). The investigation of participants was carried out at the Neurology department of the University Hospital of Tübingen and the development and validation of the algorithm were performed at Kiel University (both Germany). Before the assessments all participants gave written consent.

All participants were examined by a movement disorder specialist (WM). Participants without orthopedic problems and capable of walking without aids were included. Exclusion criteria were deep brain stimulation and Mini Mental State Examination (MMSE) score <24. Table 1 provides the demographic and clinical details of the participants. All participants were equipped with the Mobility Lab system (APDM, INC., Portland, Oregon) including 3D accelerometers (±16 g) and 3D gyroscopes (±2,000°/s), with 128 samples/s (*f*_*s*_). Participants were then asked to perform daily-like activities, such as moving around in the hospital, climbing stairs, sitting, standing, making coffee, ironing clothes and brushing teeth during an assessment period of 90–180 min (41, 42). During the entire standardized process, the participants were video-recorded with a camera (Sony, resolution 1,920 × 1,080 pixels, frame rate of 50 samples/s). The video was mostly collected within one continuous session and rest periods were also included in the analysis.

**Table 1:** Demographic and clinical data of the training and test groups.

	**PD patients**	**Older adults**
**TRAINING GROUP**		
N (females)	4 (3)	1 (0)
Age (years)	64 (18)	58 (0)
MDS-UPDRS III (0–132)	37 (2)	1 (0)
H&Y (0–5)	3 (1)	0 (0)
LED (mg)	353 (420)	0 (0)
**TEST GROUP**		
N (females)	21 (11)	11 (5)
Age (years)	68 (6)	62 (9)
MDS-UPDRS III (0–132)	29 (13)	2 (4)
H&Y (0–5)	2 (1)	0 (0)
LED (mg)	904 (611)	0 (0)

*Data are shown as mean ± standard deviation, except gender. H&Y, Hoehn & Yahr; LED, Levodopa equivalent dose (40); MDS-UPDRS III, motor part of the revised Unified Parkinson's disease (PD) Rating scale*.

Videos were evaluated by two independent clinical observers (LH and EW) to identify PT episodes and to estimate directions and durations of each PT. The clinical observers noted when a stand-to-sit or sit-to-stand movement occurred. The start of a PT episode was defined by a forward bending of the lower back. The PT episode ended when the backward movement of the lower back stopped and the participant fully sat down (in stand-to-sit) or stood up (in sit-to-stand). The PT duration was estimated using a watch and presented in full seconds. Periods during which the participant was out of the camera sight (about 5% of the total number of PTs) were discarded. The mean number of PTs measured per study participant was 33.

### Algorithm development and validation

**Figure 1:**
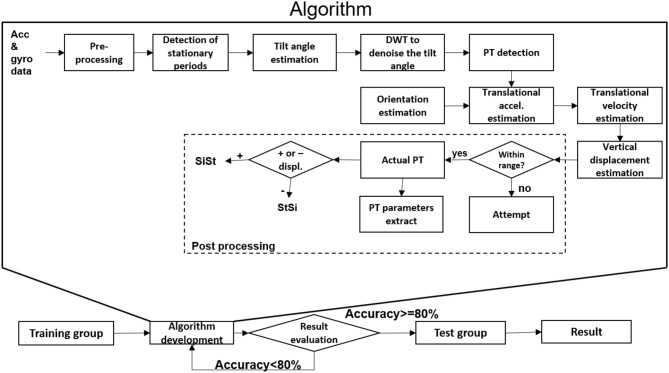
Algorithm development and validation steps for postural transition (PT) detection. Acc, accelerometer; accel, acceleration; DWT, discrete wavelet transform; gyr, gyroscope; SiSt: sit-to-stand, StSi: stand-to-sit; +/-, positive/negative.

**Figure 2:**
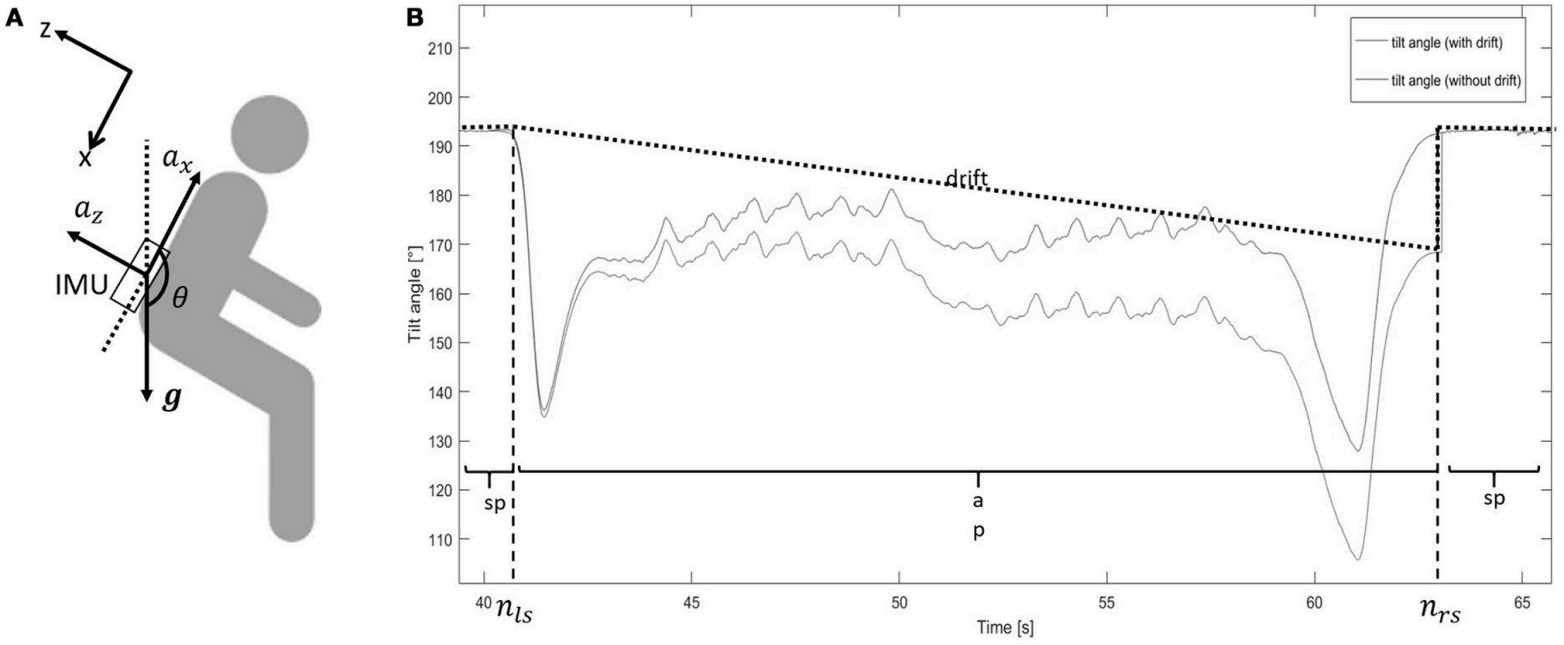
**(A)** Sensor location. The upper left *x*/*z* axis represents the reference frame (gravity, ***g***). *a*_*x*_, acceleration in vertical direction (*x*-axis); *a*_*z*_, acceleration in anterior-posterior direction (*z*-axis); IMU, inertial measurement unit; θ, tilt angle. **(B)** Tilt angle estimation from sit-to-stand and stand-to-sit movements. The blue line shows the tilt angle estimation based on integration. The black dotted line shows the drift of the integration. The red line shows the tilt angle estimation after removing the drift. *ap*, active period; *sp*, stationary period; *n*_*ls*_, latest time index of the previous stationary period; *n*_*rs*_, earliest time index of the following stationary period. The step change at *n*_*rs*_ for the “tilt angle (with drift)” line is caused by the accumulative integration error of the medio-lateral gyroscope signal. The tilt angle at that point changes from “estimated by gyroscope” to “estimated by accelerometer”.

Data obtained from the lower back IMU was used for the analysis (**Figure 2A**). First, we divided available datasets into an algorithm training dataset consisting of four PD patients (two with dyskinesia) and one OA and a test dataset consisting of 21 PD patients (five with dyskinesia) and 11 OA. We used the training dataset for the development of the algorithm, which is described in the following. The test dataset was used for validation purposes. Table 1 provides demographic and clinical details of the two groups. Figure 1 graphically presents the relevant steps of the algorithm development and the validation processes.

### Algorithm development

As an overview, the accelerometer and gyroscope data were pre-processed and the stationary periods of the lower back were identified. These stationary periods were used to estimate the tilt angle with respect to the horizontal plane. They were smoothed by using discrete wavelet transformation (DWT denoise) and the start and end of the PTs were identified. The sensor orientation during the PTs was estimated using the quaternion (estimated from accelerometer and gyroscope data) to estimate the vertical displacement of the lower back. Based on the extent of vertical displacement, PTs were classified as “effective PTs” and “PT attempts,” and the direction of the PTs was defined.

#### Preprocessing

The raw data stems from accelerometers (***a***_**0**_) and gyroscopes (**ω**_**0**_). The magnetometer data was not used, because it is affected by magnetic disturbances (43, 44). Accelerometer signals were filtered with the 4th order Butterworth phaseless, recursive low pass filter (LPF) with a cut-off frequency of *f*_*c*_ = 5 *Hz* to remove electronic noise (30). That cut-off frequency was selected based on a previous study (44), mentioning that the typical frequency range for gait is between 0 and 5 Hz. The filtered accelerometer signal and the gyroscope signal were called ***a***(*n*) = {*a*_*i*_} and **ω**(*n*) = {ω_*i*_}, respectively. The index *i* ∈ {*x, y, z*} indicates the direction (*x, y*, or *z* axis). The acceleration magnitude (|*a*(*n*)|) was extracted from the raw accelerometer data according to (Equation 1):

(1) $$\begin{array}{l} |a\left(n\right)|=\sqrt{\sum_{i=\left\{x,y,z\right\}}{a_{i}\left(n\right)}^{2}} \end{array}$$

*n* is the discrete time index of the data sampled at *f*_*s*_ = 128 *Hz*.

From the acceleration magnitude, the short-term acceleration mean (μ_*a*_(*n*)) (Equation 2) and variance ($\sigma_{a}^{2}\left(n\right)$) (Equation 3) were extracted, with *N* chosen to be 128:

(2) $$\begin{array}{l} \mu_{a}\left(n\right)=\frac{1}{N}\sum_{i=0}^{N-1}|a\left(n-i\right)| \end{array}$$

(3) $$\begin{array}{l} \sigma_{a}^{2}\left(n\right)=\frac{1}{N}\sum_{i=0}^{N-1}{\left(|a\left(n-i\right)|-\mu_{a}\left(n-i\right)\right)}^{2} \end{array}$$

The similar short-term estimations were computed for the gyroscope signal, to get the gyroscope magnitude |ω(*n*)|, the short-term gyroscope mean μ_ω_(*n*) and variance $\sigma_{\omega }^{2}\left(n\right)$. Whenever $\sigma_{\omega }^{2}\left(n\right)<10^{-5}\frac{m}{s^{2}}$, the gyroscope bias (***b***_**ω**_) was found according to (Equation 4). This threshold was selected based on the observations on the training dataset.

(4) $$\begin{array}{l} b_{\omega }\left(n\right)=\{\begin{array}{c} \omega \left(n\right),\text{ if }\sigma_{\omega }^{2}\left(n\right)<10^{-5}\frac{m}{s^{2}}\\ b_{\omega }\left(n-1\right),\text{ else} \end{array} \end{array}$$

The gyroscope bias was removed from the gyroscope signal according to (Equation 5):

(5) $$\begin{array}{l} \tilde{\omega }\left(n\right)=\omega \left(n\right)-b_{\omega }\left(n\right) \end{array}$$

In the following sections, to reduce the complexity of the equations, the index *n* will be removed except when the time indices of two quantities are different.

#### Detection of stationary periods

Stationary periods (*sp*) were defined as the episodes when the lower back of the participant was almost not moving and not rotating. While previous research has set threshold values based on mathematical approaches or estimations (45), we used thresholds determined based on the training data set. The period was defined as a stationary period (*sp*) when $|a|<0.05\frac{m}{s^{2}}$ and $\sigma_{a}^{2}<0.01\frac{m2}{s^{4}}$ and $\sigma_{\omega }^{2}<0.01\frac{1}{s^{2}}$. Otherwise, the period was considered as an active period (*ap*).

#### Tilt angle estimation

We assumed that the tilt angle of the lower back during PTs was only around the medio-lateral axis (M/L) of the body. As shown in Figure 2A, during a *sp*, accelerometers measure only gravity (*g*), whose component (*g*_*z*_, *g*_*x*_) could be used to estimate the tilt angle during *sp θ*_*sp*_(Equations 6–8):

(6) $$\begin{array}{l} g_{x}=a_{x} \end{array}$$

(7) $$\begin{array}{l} g_{z}=a_{z} \end{array}$$

(8) $$\begin{array}{l} \theta_{sp}=\{\begin{array}{c} atan2\left(g_{z},g_{x}\right),\;if\;g_{z}\geq 0\\ atan2\left(g_{z},g_{x}\right)+2\pi ,\;if\;g_{z}<0 \end{array} \end{array}$$

Tilt angle during *ap* (θ_*ap*_) was estimated as follows (Figure 2B):

- integrate the angular velocity around the medial lateral axis (ω_*y*_) (the 2nd component of the gyroscope signal without bias $\tilde{\omega }$), from the latest time index (*n*_*ls*_) of the previous stationary period, to the earliest time index (*n*_*rs*_) of the following stationary period, to get the tilt angle with drift (θ_*ap*+*d*_) (Equations 9, 10):
  (9) $$\begin{array}{l} \theta_{ap+d}\left(n_{ls}+1\right)=\theta_{sp}\left(n_{ls}\right)+\omega_{y}\left(n_{ls}\right)*\frac{1}{f_{s}} \end{array}$$
  (10) $$\begin{array}{l} \theta_{ap+d}\left(n+1\right)=\theta_{ap+d}\left(n\right)+\omega_{y}\left(n\right)*\frac{1}{f_{s}} \end{array}$$

- estimate the drift (*d*) from *n*_*ls*_ to *n*_*rs*_, according to (Equations 11, 12):
  (11) $$\begin{array}{l} d_{\theta }\left(n\right)=k_{\theta }*\left(n-n_{ls}\right)+\theta_{sp}\left(n_{ls}\right) \end{array}$$

with

(12) $$\begin{array}{l} k_{\theta }=\frac{\theta_{ap+d}\left(n_{rs}\right)-\theta_{sp}\left(n_{rs}\right)}{n_{rs}-n_{ls}} \end{array}$$

- estimate θ_*ap*_, which was the tilt angle without drift, according to (Equation 13):

(13) $$\begin{array}{l} {\theta_{ap}=\theta }_{ap+d}-d_{\theta } \end{array}$$

The tilt angle (θ), consisting of θ_*sp*_ and θ_*ap*_, was used for further analysis.

#### Discrete wavelet transformation (DWT) to denoise the tilt angle (θ)

**Figure 3:**
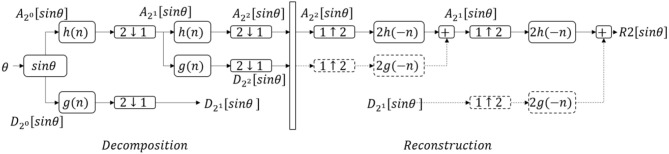
The denoising process with discrete wavelet transform. θ, tilt angle; $A_{2^{j}}\left[sin\theta \right]$, low frequency components; $D_{2^{j}}\left[sin\theta \right]$, high frequency components with *j*, number of iteration; *h*(*n*), low pass filter; *g*(*n*), high pass filter; 2↓1, downsample by half; 1↓2, upsample by double; *Rj*[*sinθ*], denoised signal after *j* iterations. Dash lines mean suppressing the components to zero. This figure shows an example of the denoising process with two iterations.

DWT was used to remove the integration drift and movement artifacts (30). To scale θ from −1 to 1, sinθ was calculated, then denoised using DWT (Figure 3). sinθ was passed through a LPF [with *h*(*n*) as the impulse response] and high pass filter (HPF) [with *g*(*n*) as the impulse response], then both were down-sampled by half (2↓1). sinθ was split into the low-frequency component ($A_{2^{1}}\left[sin\theta \right]$), and the high-frequency component ($D_{2^{1}}\left[sin\theta \right]$). This step is called one deconstruction step. $A_{2^{1}}\left[sin\theta \right]$ was deconstructed again, split into $A_{2^{2}}\left[sin\theta \right]$ and $D_{2^{2}}\left[sin\theta \right]$. *h*(*n*) and *g*(*n*) were chosen by the Coiflet order 5 (46). The process was continued similarly on the lowest-frequency component to get $A_{2^{j}}\left[sin\theta \right]$ and $D_{2^{j}}\left[sin\theta \right]$, with *j* is the number of iteration.

In the reconstruction step, all high-frequency components were suppressed to zero (Equation 14):

(14) $$\begin{array}{l} D_{2^{j}}\left[sin\theta \right]=\{\begin{array}{c} 0,\;if\;j>0\\ D_{2^{j}}\left[sin\theta \right],\;if\;j=0 \end{array} \end{array}$$

$A_{2^{0}}\left[sin\theta \right]$ was upsampled (1↑2) by double, to get the denoised signal (*Rj*[*sinθ*]). This process is called reconstruction.

In this study, *j* was taken to be three times (to get *R*3[sinθ]) and 10 times (to get *R*10[sinθ]) (based on the training results). The symbol *R*3[sinθ] describes the aforementioned process “deconstruct the signal sinθ three times; suppress the high-frequency components; reconstruct with the lowest frequency component.” The symbol *R*10[sinθ] describes “deconstruct the signal sinθ 10 times; suppress the high-frequency components; reconstruct with the lowest frequency component.” The difference between *R*3[sinθ] and *R*10[sinθ], called *tilt*_*denoise* (*tilt*_*denoise* = *R*3[sinθ]−*R*10[sinθ]), was used for further analysis.

#### Postural transition (PT) detection

**Figure 4:**
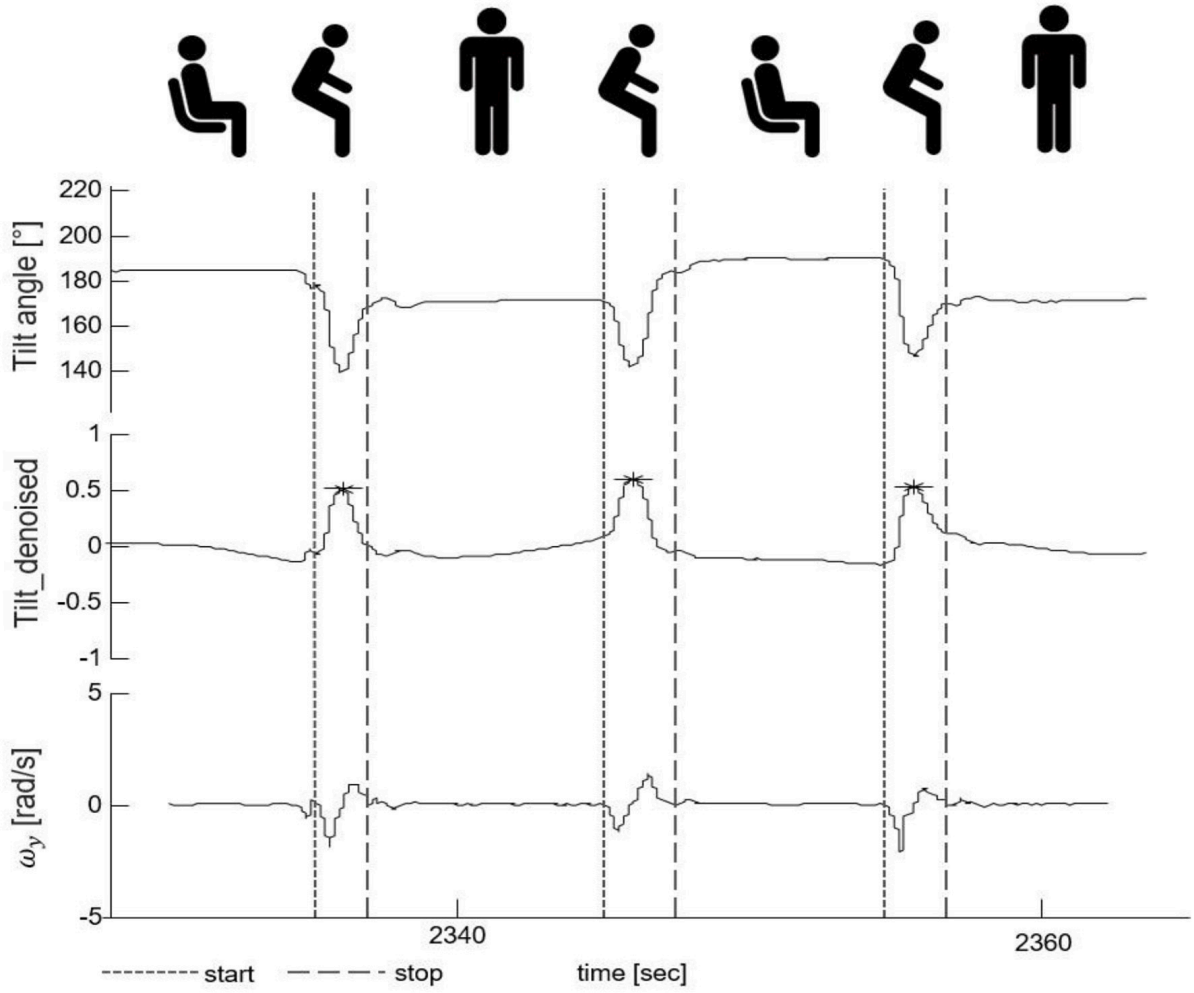
Postural transition (PT) detection and PT start/end identification. The upper part shows the tilt angle. The middle part shows the denoised tilt signal used for PT detection. The lower part presents the medio-lateral-axis gyroscope signal (ω_*y*_) whose zero-crossings were used for the identification of the start and the end of a PT. The fine-dashed vertical line indicates the start and the gross-dashed line the end of the respective PTs. The star symbols indicate the PT events with magnitude and prominence >0.1.

The peaks of the *tilt*_*denoise* signal with magnitude and prominence >0.1 were defined as PT events (indicated by a star symbol, Figure 4). In the following parts, they were classified into either “effective PTs” (i.e., the participant was considered to perform a complete standing up or sitting down movement) or “PT attempts” (i.e., the participant was considered not to perform a complete PT, e.g., forward and backwards body motion).

The zero-crossing method was used to define the beginning and the end of a PT in the gyroscope signal (47) (indicated by vertical lines, Figure 4). The beginning of a PT was defined as the first zero crossing point of the medio-lateral angular velocity (ω_*y*_) on the left side of the PT event, with negative slope. The end of a PT was defined as the zero crossing point of ω_*y*_ on the right side of the PT event, again with negative slope.

#### Orientation estimation of the sensor with respect to the earth frame

**Figure 5:**
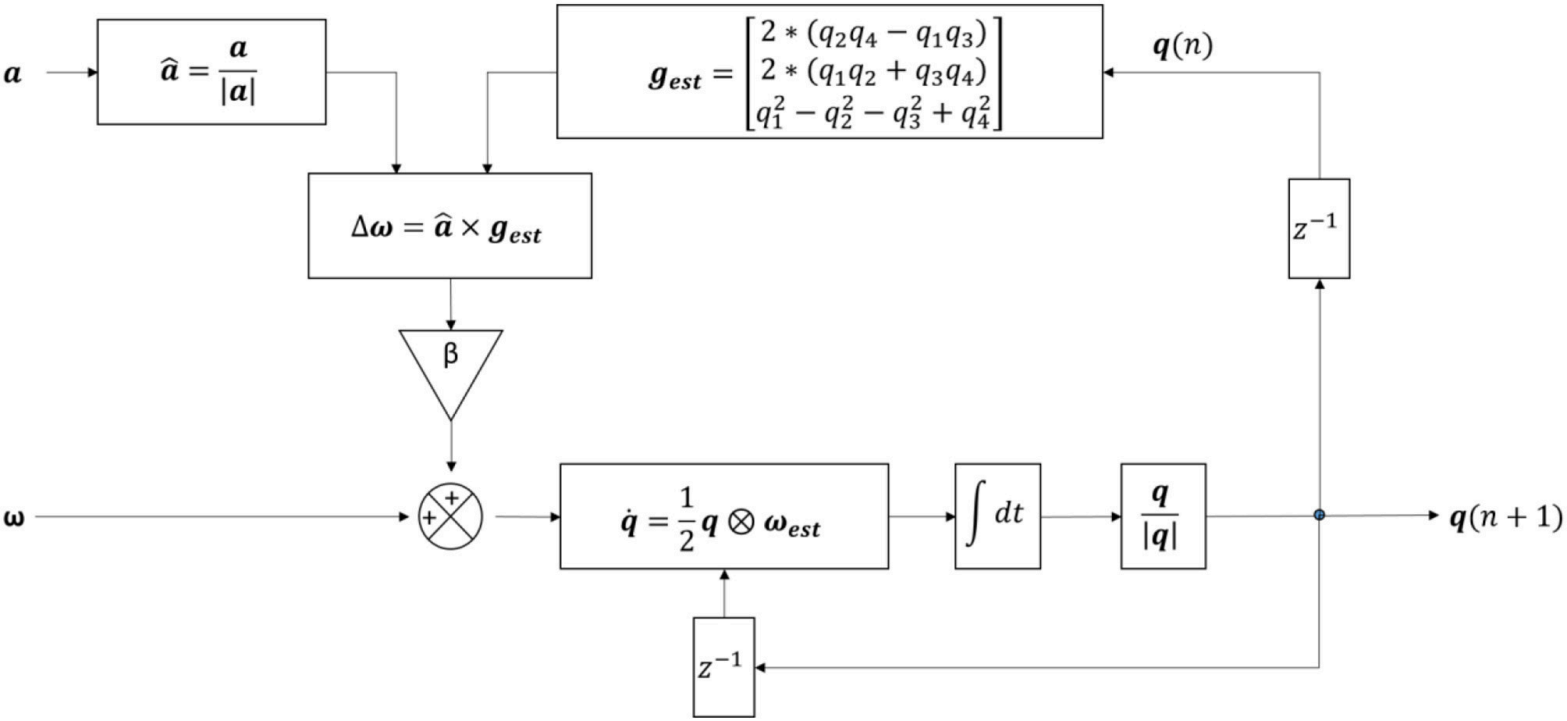
Structure of the algorithm for the detection of postural transitions using six degree-of-freedom IMU sensor fusion. ***a***, pre-processed accelerometer signal; $\hat{a}$, normalized acceleration; |***a***|, magnitude of the acceleration; **β**, feedback coefficient; ***g***_***est***_, estimated gravity; ∫dt, integration symbol; $\tilde{\omega }$, gyroscope signal with no bias; **ω**, feedback fixation of $\tilde{\omega }$; **ω**_***est***_, re-estimated $\tilde{\omega }$; *q*_1_, *q*_2_, *q*_3_, *q*_4_, four components of the quaternion; ***q***(*n*), quaternion from the current iteration; ***q***(*n*+1), quaternion from the next iteration; $\dot{q}$, change in quaternion; ***q***, quaternion; |***q***|, quaternion magnitude; *z*^−1^, time shift operator.

A 6DOF fusion algorithm was used to represent the orientation of the sensor with respect to the earth frame in quaternion ***q*** (48) (Figure 5). ***q*** has the form of [*q*_1_ *q*_2_ *q*_3_ *q*_4_], with the initial orientation value $q\left(0\right)=\left[\frac{1}{\sqrt{2}}\;0\;\left(-\frac{1}{\sqrt{2}}\right)\;0\right]\;$once the *sp* is firstly detected. Gravity in the sensor frame (***g***_*est*_) was estimated using ${\text{g}}_{\text{e}s\text{t}}=\left[\begin{array}{c} 2^{*}\left(q_{2}q_{4}-q_{1}q_{3}\right)\\ 2^{*}\left(q_{1}q_{2}+q_{3}q_{4}\right)\\ q_{1}^{2}-q_{2}^{2}-q_{3}^{2}+q_{4}^{2} \end{array}\right]$. The preprocessed acceleration vector (***a***) was normalized to $\hat{a}=\frac{a}{\left|a\right|}.$ The angular velocity (**ω**_*est*_) was re-estimated (calculated by $\omega_{\text{est}}=\tilde{\omega }+\beta △\omega$) with △**ω** is the feedback fixation of $\tilde{\omega }$ (calculated by $\omega =\hat{a}\times g_{est}$, with × is the cross product) and β is the coefficient (which is 0 during *ap*, and 0.5 during *sp*). The quaternion change ($\overset{{}^{\circ}}{q}$) was computed $\dot{q}=\frac{1}{2}q⊗\omega_{est}$ (with ⊗ is the quaternion multiplication) and used to update the current quaternion using the following formula (Equation 15):

(15) $$\begin{array}{l} q\left(n+1\right)=q\left(n\right)+\dot{q}\left(n\right)*\frac{1}{f_{s}} \end{array}$$

***q*** values were normalized $\tilde{\text{q}}=\frac{\text{q}}{\left|\text{q}\right|}$ before being used for further analysis.

#### Translational acceleration estimation (*a_T_*)

***a*** was converted to the earth frame (^***E***^***a***) (Equation 16):

(16) $$\begin{array}{l} \left[0{\;}^{E}a\right]=\tilde{q}⊗\left[0\;a\right]⊗{\tilde{q}}^{*} \end{array}$$

where ${\tilde{q}}^{*}$ is the quaternion conjugation of the quaternion (Equation 17)

(17) $$\begin{array}{l} {\tilde{q}}^{*}=[{\tilde{q}}_{1}\;-{\tilde{q}}_{2}\;-{\tilde{q}}_{3}\;-{\tilde{q}}_{4}] \end{array}$$

The gravity component (^***E***^***g***), which equals [0 0 1] in the earth frame, was eliminated from the ^***E***^***a*** to get only translational acceleration (***a***_***T***_) (Equation 18):

(18) $$\begin{array}{l} a_{T}{=}^{E}a{-}^{E}g \end{array}$$

#### Translational velocity (*v*_*T*_) and vertical displacement (*d*_*Z*_) estimation

During *sp*, ***v***_***T***_ was set to zero. During *ap*, translational velocity with drift (***v***_***T*+*d***_) was obtained by integrating *a*_*T*_ (Equation 19):

(19) $$\begin{array}{l} v_{T+d}=a_{T}.\frac{1}{f_{s}} \end{array}$$

Similar to the aforementioned part “Tilt angle estimation,” with ***v***_***T***_ during *sp* being zero, the drift (***d***_***v***_) was estimated (Equations 20, 21) and removed from ***v***_***T*+*d***_, to get ***v***_***T***_:

(20) $$\begin{array}{l} d_{v}\left(n\right)=k_{v}n-k_{v}n_{ls} \end{array}$$

(21) $$\begin{array}{l} \text{with }k_{v}=\frac{v_{T+d}\left(n_{rs}\right)}{n_{rs}-n_{ls}} \end{array}$$

During the *ap* period, **v**_**T**_ along the vertical axis (*v*_*z*_, which is the 3rd component of **v**_**T**_) was integrated to obtain the vertical displacement (*d*_*z*_) (Equation 22):

(22) $$\begin{array}{l} d_{z}=v_{z}.\frac{1}{f_{s}} \end{array}$$

The difference of *d*_*z*_ between the end and the beginning of each PT events (△*d*_*z*_) was calculated and was used for further analysis. A threshold was set to △*d*_*z*_ = 0.1*m* to differentiate between effective PTs and PT attempts. PT attempts were excluded from the final result. Regarding the direction of the PTs, positive △*d*_*z*_ was defined as a sit-to-stand, and negative △*d*_*z*_ as a stand-to-sit movement. The duration of the PT was defined as the time between the beginning and the end of the PT.

### Statistical analysis

Analyses were conducted using JMP 11.1.1 software (SAS Institute GmbH, Böblingen, Germany). Demographic data of the training group and the validating group is presented with mean and standard deviation (Table 1). Intraclass correlation (ICC) was used to evaluate the agreements between two clinical observers (EW and LH). Contingency table was used to calculate the sensitivity, accuracy and positive predictive value for PT detection. 888 true positive PTs were used for the inference statistics. Student *t*-test was performed to detect PT duration differences between PD patients and OA for both the algorithm and the video reference. PT direction (SiSt or StSi) of the true positive PTs was compared between the algorithm and the video reference. The difference of the PT duration, estimated by the algorithm and the video reference, and the 95% confidence interval (CI) were also computed.

## Results

The ICC of the two clinical observers regarding PT detection was 0.99. Total number of detected PTs were 1,001 and 1,064 for clinical raters and the algorithm, respectively. The accuracy of the algorithm to detect PTs reached 82% for PD patients without dyskinesia, 47% for PD patients with dyskinesia and 85% for OA (Table 2). The overall accuracy regarding the PT direction was 98%.

**Table 2:** Validation values for detection of postural transitions (PTs) derived from the test group.

**Groups**	**Acc**.	**Sens**.	**PPV**	**PT detected by the algorithm**	**PT detected by the clinical observers**	**True positive PT**	**False positive PT**	**False negative PT**
All participants (*N* = 32)	77	89	83	1,064	1,001	888	176	113
OA (*N* = 11)	85	98	86	307	273	265	42	6
PD without dyskinesia (*N* = 16)	82	89	90	595	599	533	61	67
PD with dyskinesia (*N* = 5)	47	69	55	162	129	89	73	40

*Values are calculated on a sample-by-sample basis. Acc., accuracy; NPV, negative predictive value; PD, Parkinson's disease; OA, older adults; PPV, positive predictive value; Sens, sensitivity*.

**Figure 6:**
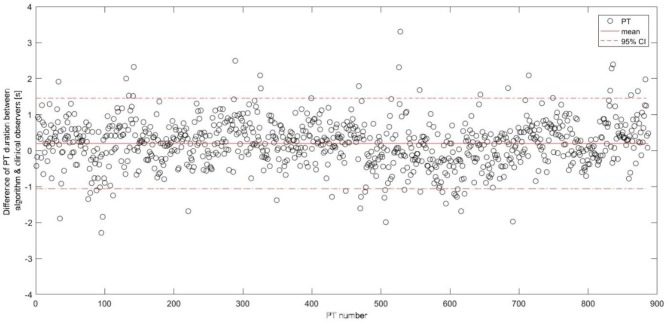
Differences between the postural transition duration estimation including the mean and the 95% confidence interval represented as horizontal lines. PT, postural transition; CI, confidence interval.

The algorithm yielded a mean (SD) PT duration of 1.96 (0.72)s for PD patients and 1.74 (0.43)s for OA, with 0.22 s PT duration difference (*p* < 0.001) between the groups showing a comparable value with the video reference (0.26 s, *p* < 0.001). Figure 6 shows the difference of the PT duration, estimated by the algorithm and the video reference including the 95% CI. The mean duration difference between the algorithm and the video reference was 0.20 s, with a 95% CI between −1.06 s and 1.45 s.

## Discussion

In this paper, we present an algorithm for successful PT detection and quantification from a single wearable sensor on the lower back in a home-like environment. The algorithm was developed and tested with PD patients with and without dyskinesia and OA, and the results were compared against video observation.

The introduced algorithm yielded accuracies of 82% for PD patients without dyskinesia, 47% for PD patients with dyskinesia and 85% for OA. To our knowledge, this is the first validated PT detection algorithm for PD patients and OA based on home-like-environment data using one sensor fixed to the lower back.

Previous research (27, 29, 31, 49) has reported higher detection accuracies, however the studies either estimated PTs in healthy adults or during scripted laboratory protocols/supervised conditions, which differs to daily-life conditions.

Equally, the studies investigating PT occurrences in home-like conditions (50, 51) showed very promising results for healthy OA, frail OA and for post-stroke patients. Nevertheless, the groups and the IMU localization differ; therefore, accuracy values should be compared with caution.

With regards to PD patients, one study (52) reported high accuracy values for the detection and evaluation of PTs in healthy individuals (94%) and PD patients (87%) in home-like conditions using a fuzzy classifier, but the definition of PT was not specified to sit-to-stand and stand-to-sit movements and multiple sensors were used.

We argue that data obtained from the lower back is preferable, as the position is closer to the center of mass (53), and lower back-derived algorithms for PT-“associated” movements, such as walking (33, 54, 55) and turning (32, 47, 56), show excellent accuracy values. The inclusion of PD patients and healthy OA in the validation process supplemented the robustness of the proposed algorithm yielding in excellent accuracy regarding the PT direction identification (98%), for further investigations.

This study used discrete wavelet transform to remove noise and to deal with integration errors in tilt angle estimation from the gyroscope bias (30, 57). The tilt angle was estimated by integrating the gyroscope and this computation step avoids inverse trigonometric functions, resulting in faster computation speed. This wavelet technique also enhances the height of PT peaks in the tilt angle pattern, suppresses the superfluous peaks (i.e., the peaks in the pattern produced by other activities) and consequently increases the sensitivity of PT detection. The inclusion of the orientation estimation (by using the quaternion) and the vertical displacement led to a substantial improvement of accuracy values compared to vertical acceleration and velocity. Specifically, this approach reduced, compared to the mentioned vertical acceleration and velocity approach, the number of false positive PTs due to erroneous detection of trunk movements (which regularly occur during daily activity movements, such as ironing). In summary, we feel that we can provide here a mature “hypothesis-derived” algorithm for PT detection in PD patients and OA, however with potential of further improvement (see also below).

Although our results are promising, still more work is necessary, particularly with the definition and validation of quantitative PT parameters. Most promising candidate parameters are, in our view, flexion and extension tilt angles of the lower back during PTs (58), as well as flexion and extension angular velocities (59).

With regard to the dyskinetic PD patients, the algorithm was not sufficiently accurate. The main reason is that these patients were constantly moving, which prevented the algorithm from detecting stationary periods sufficient to estimate a correct vertical displacement (50, 60). One option to overcome this issue, at least in patients that are not continuously dyskinetic, is to use algorithms for dyskinesia detection (41, 61) and to remove dyskinetic phases before the PT evaluation. For the evaluation of PTs during dyskinetic phases, an additional barometric pressure sensor could be included as it allows the accurate estimation of vertical displacement (50, 62).

Mean PT duration of 1.89 s is comparable to other studies including PD patients (31). The mean PT duration difference between the algorithm and the video reference was 0.20 s and comparable to (27). The difference is partly explained by the video reference measurement. Measuring in full seconds may increase the mean PT duration difference and consequently the confidence intervals between the IMU-based and video reference durations (Figure 6). Moreover, it is often difficult for clinical observers to differentiate between an effective PT and a PT attempt. For example, we experienced that especially slow and long PT attempts were occasionally classified as a single PT *attempt* by the clinical observers, while being identified as two PTs (i.e., a sit-to-stand and stand-to-sit movement) by the algorithm. Such a misclassification leads consequently to two false positives and this misinterpretation explained 60% of the false positives in our validation group. Furthermore, we have still some false positive PT detections from the activities involving the leaning forward and backward of the upper body, such as during ironing clothes, picking objects, and tying shoelaces. False negative PT detections seem to originate mainly from rigid sit-to-stand and stand-to-sit episodes in severely bradykinetic PD patients (63). Those specific patterns need to be further investigated in order to reduce the false detection, hence improve the accuracy of the algorithm.

## Conclusion

We present here an algorithm for PT detection in PD patients and OA who all performed purposeful PTs in a home-like environment. The validation values for PD patients and OA justify, in our view, the use of the algorithm in pilot studies performed in clinical and home-based settings. Our algorithm needs further validation particularly with regard to PT quantification, to provide, e.g., validated PT angle-related parameters. Moreover, further exploration is required particularly in specific subgroups performing “unusual” movements (here: PD patients suffering from dyskinesia), e.g., within collaborations of algorithm developing research groups including cross-validation approaches.

## Author contributions

MP, GS, and WM were responsible for the conception and design of the study. WM contributed to the data acquisition. MP, EW, ME, L-MB, JF, DB, MH, LH, CH, GS, and WM were involved in the analysis and interpretation of data. MP, ME, EW, CH, and WM drafted the first version of the article. All authors revised it critically for important intellectual content. All authors gave final approval of the version to be submitted.

### Conflict of interest statement

The authors declare that the research was conducted in the absence of any commercial or financial relationships that could be construed as a potential conflict of interest.
